# Immunomodulation of endothelial cells induced by macrolide therapy in a model of septic stimulation

**DOI:** 10.1002/iid3.518

**Published:** 2021-10-12

**Authors:** Stéphanie Pons, Eden Arrii, Marine Arnaud, Maud Loiselle, Juliette Ferry, Manel Nouacer, Julien Lion, Shannon Cohen, Nuala Mooney, Lara Zafrani

**Affiliations:** ^1^ Human Immunology, Pathophysiology, Immunotherapy (HIPI), INSERM U976 Université de Paris Paris France; ^2^ Department of Anesthesiology and Critical Care, Pitié‐Salpêtrière Hospital, GRC 29, AP‐HP, DMU DREAM Sorbonne University Paris France; ^3^ Medical Intensive Care Unit AP‐HP, Saint‐Louis Teaching Hospital Paris France

**Keywords:** antibiotics, endothelium, immunoregulation, septic shock, inflammation

## Abstract

**Objectives:**

Sepsis is defined as the host's inflammatory response to a life‐threatening infection. The endothelium is implicated in immunoregulation during sepsis. Macrolides have been proposed to display immunomodulatory properties. The goal of this study was to analyze whether macrolides can exert immunomodulation of endothelial cells (ECs) in an experimental model of sepsis.

**Methods:**

Human ECs were stimulated by proinflammatory cytokines and lipopolysaccharide before exposure to macrolides. ECs phenotypes were analyzed by flow cytometry. Cocultures of ECs and peripheral blood mononuclear cells (PBMCs) were performed to study the ECs ability to alter T‐cell viability and differentiation in the presence of macrolides. Soluble factor production was assessed.

**Results:**

ECs act as non‐professional antigen presenting cells and expressed human leukocyte antigen (HLA) antigens, the adhesion molecules CD54, CD106, and the coinhibitory molecule CD274 after septic stimulation. Incubation with macrolides induced a significant decrease of HLA class I and HLA class II HLA‐DR on septic‐stimulated ECs, but did not alter either CD54, CD106, nor CD274 expression. Interleukin‐6 (IL‐6) and IL‐8 production by stimulated ECs were unaltered by incubation with macrolides, whereas Clarithromycin exposure significantly decreased IL‐6 gene expression. In cocultures of septic ECs with PBMCs, neither the proportion of CD4 ^+ ^, CD8 ^+ ^T nor their viability was altered by macrolides. T‐helper lymphocyte subsets Th1, Th17, and Treg polarization by stimulated ECs were unaltered by macrolides.

**Conclusion:**

This study reports phenotypic and gene expression changes in septic‐stimulated ECs exposed to macrolides, without resulting in altered immunogenicity of ECs in co‐cultures with PBMCs. In vivo studies may help to further understand the impact of macrolide therapy on ECs immune homeostasis during sepsis.

## Graphical abstract

This study reports phenotypic and gene expression changes in septic‐stimulated microvascular endothelial cells after exposure to macrolides. Although a significant decrease in cell‐surface human leukocyte antigen (HLA) class I and HLA‐DR expression and in interleukin‐6 (IL‐6) gene expression was observed, these modifications did not result in altered functional response following endothelial cells' interaction with peripheral blood mononuclear cells (PBMCs) regarding T‐lymphocyte viability, cytokine production, or CD4 ^+^ ‐T differentiation.

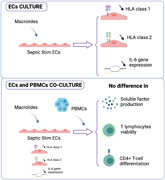

## 1 INTRODUCTION

Sepsis is defined as the host's inflammatory response to a life‐threatening infection.^1^ Sepsis induces immune dysregulation with the initiation of a strong inflammatory response. This occurs secondary to the binding of pathogen‐associated molecular patterns (PAMPs) and damage‐associated molecular patterns (DAMPs) to pattern recognition receptors such as Toll‐like receptors (TLRs) and Nod‐like receptors (NLRs) and contributes to organ failure.^2^ Immune suppression and exhaustion occur in parallel with hyper‐inflammation and predispose patients to secondary infections, reactivation of latent viruses^3^ and late deaths.^4^ Septic shock is the most important cause of mortality in intensive care unit (ICU), despite many improvements in care that have been made in the past 10 years. Currently, the most efficient weapon to combat bacterial infections is antibiotic therapy. Macrolides are a class of bacteriostatic antibiotics acting by inhibiting bacterial protein synthesis. They have a broad spectrum of activity against many Gram‐positive and some Gram‐negative bacteria. In critically ill patients, guidelines recommend a macrolide combined with a B‐lactam in patients with community acquired pneumonia (CAP) to ensure coverage of typical pathogens, such as *Streptococcus pneumoniae*, and atypical pathogens as *Mycoplasma, Legionella*, and *Chlamydophila* species.^5^ Next to their antimicrobial activity, more and more elements suggest that some antibiotic classes, mostly macrolides, may display intrinsic immunomodulatory properties.^6^ In CAP, macrolides associated with B‐lactams were observed to decrease mortality compared with B‐lactam monotherapy and to temper inflammatory responses.^7^, ^8^ In patients with CAP unresponsive to treatment after 72 h, those receiving macrolides had lower concentrations of cytokines (Interleukin [IL]−6 and tumor necrosis factor [TNF]‐α) in bronchoalveolar lavage (BAL) fluid, and regained clinical stability more quickly than patients receiving other antibiotic regiments.^9^ In mouse models of lethal pneumonia, it has been reported that macrolides could significantly modify the surface phenotype of neutrophils including altered expression of CD86, Major Complex of Histocompatibility class II antigens (MHC II) and of Programmed Death‐1 in lymphocytes.^10^ Moreover, immunomodulatory effects of macrolides have been studied in acute respiratory distress syndrome (ARDS), in which massive bilateral inflammation of the lungs secondary to pneumonia or another local or systemic condition, results in alveolar flooding and respiratory failure, with a high chance of death. Simonis et al. performed a secondary analysis of a large prospective observational study of ARDS patients in ICU. They analyzed the effects of low‐dose macrolides administered for nonantibiotic purposes on 30‐day mortality. They included 873 patients with ARDS, of whom 158 received macrolides for nonantibiotic purposes, and found a reduced 30‐day mortality in the macrolide group (22.8% vs. 31.6%; odds ratio 0.64 [interquartile range, 0.43–0.96], *p* = .03), confirmed in a propensity score matched cohort.^11^ Moreover, Walkey et al. performed a secondary analysis of a multicenter, randomized controlled trial, including patients with acute lung injury. Forty‐seven out of 235 patients received a treatment with macrolides for a median duration of 4 days. In this study, after adjusting for potentially confounding covariates, use of macrolides was associated with a lower 180‐day mortality (hazard ratio: 0.46; 95% confidence interval [CI], 0.23–0.92; *p* = .028) and a shorter time to successful discontinuation of mechanical ventilation (hazard ratio: 1.93; 95% CI, 1.18–3.17; *p* = .009).^12^

The vascular endothelium has a crucial role in several physiological processes: blood fluidity, vasomotor tone regulation, osmotic balance, vascular barrier function, and immune response.^13^, ^14^ The vascular endothelial cells (ECs), because of their localization, are the first to interact with the microbial components present in the blood. They can detect danger signals from pathogens, by the recognition of PAMPs or DAMPs and initiate the innate immune response. Indeed, they can induce local inflammation and recruitment of immune cells including leukocytes or macrophages.^15^ Moreover, ECs constitutionally express MHC class I, displaying peptide fragments of proteins from within the cells that can activate T cells (CD8 ^+ ^‐T cells). These cytotoxic T‐cells play a critical role in the control of viral infection.^16^ Moreover, human microvascular ECs express a low level of human leukocyte antigen (HLA) class II antigens in the steady‐state and expression is highly increased under inflammatory conditions,^17^ allowing CD4 ^+ ^‐T lymphocyte activation by these cells.^18^ Previous studies revealed that HLA‐DR expressing ECs could regulate CD4 ^+ ^‐T lymphocytes by promoting Th17, Treg, and/or Th1 responses.^19^, ^20^ CD4 ^+ ^‐T lymphocytes are required for an optimal immune response to bacterial infection^21^ and have a key role in sepsis and sepsis‐induced immunosuppression.^22^, ^23^

Sepsis, as well as other diseases, induces an acute systemic inflammation leading to homeostasis dysregulation partly due to lost or inappropriate ECs functions.^24^, ^25^ Consequently, the use of macrolides during bacterial sepsis could modulate the host immune response, by modifying the EC ability to recruit and activate innate and adaptive immune cells. However, the direct effects of macrolides on the immune properties of the EC have not been explored. The goal of this study was to analyze whether or not macrolides can exert immunomodulation of ECs and alter immunoregulation during sepsis.

## 2 MATERIAL AND METHOD

### 2.1 Cell lines and culture reagents

The human dermal microvascular endothelial HMEC‐1 cell line, provided by A. Kesikli (University of Regensburg, Germany) was cultured in complete MCDB 131 medium, composed of MCDB 131 medium (Thermofisher Scientific) supplemented with 12.5% fetal bovine serum (FBS), hydrocortisone 1 μg/ml (Sigma‐Aldrich), epidermal growth factor (EGF) 10 ng/ml (BD Biosciences), 6 mM Glutamine and used between passages 12 and 20.^19^, ^20^ The hCMEC/D3 cell line was cultured in complete EndoGRO Basal Medium (Merck), composed of EndoGRO Basal Medium supplemented with 0.2% EndoGRO LS Supplement, recombinant EGF 5 ng/ml, hydrocortisone hemisuccinate 1 μg/ml, heparin sulfate 0.75 U/ml, ascorbic acid 50 μg/ml, l‐Glutamine 10 mM, 5% FBS and used between passage 30 and 40.^26^ Coculture experiments were carried out with ECs and peripheral blood mononuclear cells (PBMCs), as reported elsewhere.^19^, ^20^ Briefly, PBMCs were isolated from healthy donor blood samples (obtained in accordance with institutional regulations from the Etablissement Français du Sang) by Ficoll density gradient separation (Eurobio). PBMCs were maintained in complete RPMI 1640 medium (Thermofisher Scientific), composed of RPMI 1640 with 10% human AB serum and a final concentration of HEPES 10 mM, sodium pyruvate 1× (Eurobio), glutamine 2 mM.

### 2.2 ECs treatment by inflammatory molecules and macrolides therapy

ECs were exposed in vitro to septic stimulation for 24 h, by an association of proinflammatory cytokines increased during sepsis: Interferon γ (IFN‐γ) 15 ng/ml (R&D Systems), TNF‐α 5 ng/ml (Peprotech) and lipopolysaccharide (LPS) 100 ng/ml extracted from *Escherichia coli*. After 24 h of septic stimulation, ECs were incubated with macrolides: Spiramycin, Erythromycin, or Clarithromycin (Merck) at the indicated concentrations for another 24 h. Parallel cultures were carried out with the relevant diluent before phenotypic, quantitative polymerase chain reaction (qPCR), and functional assays.

### 2.3 Antibodies and flow cytometry

The following antibodies were used: CD54 Alexa Fluor 488 (AF488), HLA class I (HLA‐I) allophycocyanin‐cyanine 7 (APC‐Cy7), CD274 phycoerythrin‐cyanine 7 (PE‐Cy7) (Biolegend), CD106 APC and HLA class II HLA‐DR APC (BD Bioscience). In cocultures, for phenotypic analysis of CD4 ^+ ^‐T, the following antibodies were used: CD4 PE (clone RPA‐T4), IFN‐γ Fluoresceine isothiocyanate (FITC) (clone B27), HLA‐DR APC (clone G46‐6), CD3 PerCP (clone SK7), CD4 Pacific Blue (PB) (Clone RPA‐T4), CD45RA PE‐Cy7 (clone H100), CD25 PE (clone M‐A251), CD127 peridinin‐chlorophyll‐protein cyanine 5.5 (PerCP‐Cy5.5) (clone A019D5), CD54 PB (clone HCD54) (Biolegend), CD8 PB (Clone RPA‐T8) (BD Biosciences), Interleukin 17 efluor660 (eBioscience). Intracellular staining of FoxP3 was carried out with the anti‐Human Foxp3 Staining Set APC (clone 236 A/E7) (eBioscience). For one experiment, each condition was tested and analyzed in duplicate or triplicate. Flow cytometry was carried out on a FACS Canto II (BD Biosciences).

### 2.4 Cocultures assays

After septic stimulation for 24 h, ECs were either exposed to macrolides or to control conditions for 24 h, before washing and incubation in fresh medium. Cells were then washed three times and irradiated (20 Gy) to prevent further proliferation and cocultured with PBMCs at a ratio 1:1 for 7 days as described elsewhere.^19^ The supernatants of cocultures were collected after 72 h for cytokine measurement. At Day 7 of the coculture, PBMCs were stimulated by phorbol‐12‐myristate‐13‐acetate 50 ng/ml, and ionomycin 1 µM (Cell Signaling Technology) in the presence of GolgiStop (BD Biosciences) for 4 h before labeling cells to detect T lymphocytes expressing intracellular IL‐17 (CD3 ^+ ^CD8 ^− ^IL‐17^+^) or IFN‐γ (CD3 ^+ ^CD8 ^− ^IFN‐γ^+^) by flow cytometry^20^, ^27^ (Figure S1).

### 2.5 Lymphocyte viability

After EC septic‐stimulation for 24 h, ECs were either exposed to macrolides or to control conditions for 24 h, then ECs were washed and incubated in fresh medium. Cells were then cocultured with PBMCs at a ratio 1:1 for 3 days. At Day 3 of the coculture, PBMCs were incubated for 30 min at 4°C with CD3, CD4, and CD8 human antibodies. To measure apoptosis, PBMCs were incubated with Annexin V‐FITC and 7‐aminoactinomycin D (7‐AAD) (Biolegend), and analyzed by flow cytometry within 1 h.

### 2.6 Cytokines detection by enzyme‐linked immunosorbent assay (ELISA)

IL‐6, IL‐8, regulated upon activation‐normal T cell expressed and presumably secreted (RANTES), and soluble intercellular adhesion molecule (ICAM‐1s) were measured in supernatants of ECs cultures and IL‐6, IL‐8, IL‐2, and IFN‐γ in supernatants of ECs cocultures with PBMCs, using ELISA kits (BD Biosciences) and according to the manufacturer's protocol. All the samples were assayed in duplicate.

### 2.7 Real‐time polymerase chain reaction analysis

HLA‐A, HLA‐DR, IL‐6, and Glyceraldehyde‐3‐phosphate dehydrogenase (GAPDH) messenger RNA (mRNAs) were assayed using a fluorescence‐based real‐time PCR. After 24 h of septic stimulation followed by 24 h of macrolide therapy, total RNA was isolated from ECs using the TRI Reagent (Thermo Fischer Scientific) protocol. RNA was quantified using a spectrophotometer ND‐1000; Nanodrop (Thermo Fischer Scientific) and converted to complementary DNA (cDNA) (1 µg RNA/reaction) by reverse transcription (RT) using the SuperScript III First‐Strand Synthesis System for Real‐time PCR (Invitrogen Life Technologies). Real‐time PCR was performed with ViiA 7 Real‐Time PCR System (Thermo Fischer Scientific) and TaqMan gene Expression Assay (Thermo Fischer Scientific). The primers and probe sets used for this study were: IL‐6 (Hs00174131_m1), HLA‐A (Hs01058806_g1), HLA‐DR (Hs00219575_m1), and GAPDH (Hs02758991_g1). Threshold cycles (*C*_t_) were determined as the mean of duplicate determinations. The differences in relative abundances of mRNA were calculated as 2^‐∆∆Ct^.

### 2.8 Statistical analysis

Statistical analysis was performed using the GraphPad Prism software (GraphPad Software). The results are reported as mean ± standard error of the mean (SEM). The statistical significance of the data was determined using the indicated parametric or non‐parametric tests, according to the normal distribution of the data. Normality was assessed using Shapiro–Wilk test. For analysis of variance (ANOVA) multiple comparison, a Dunnett post hoc test has been conducted. A *p* < .05 was considered statistically significant.

## 3 RESULTS

### 3.1 Macrolide therapy modifies the ECs phenotype

**Figure 1:**
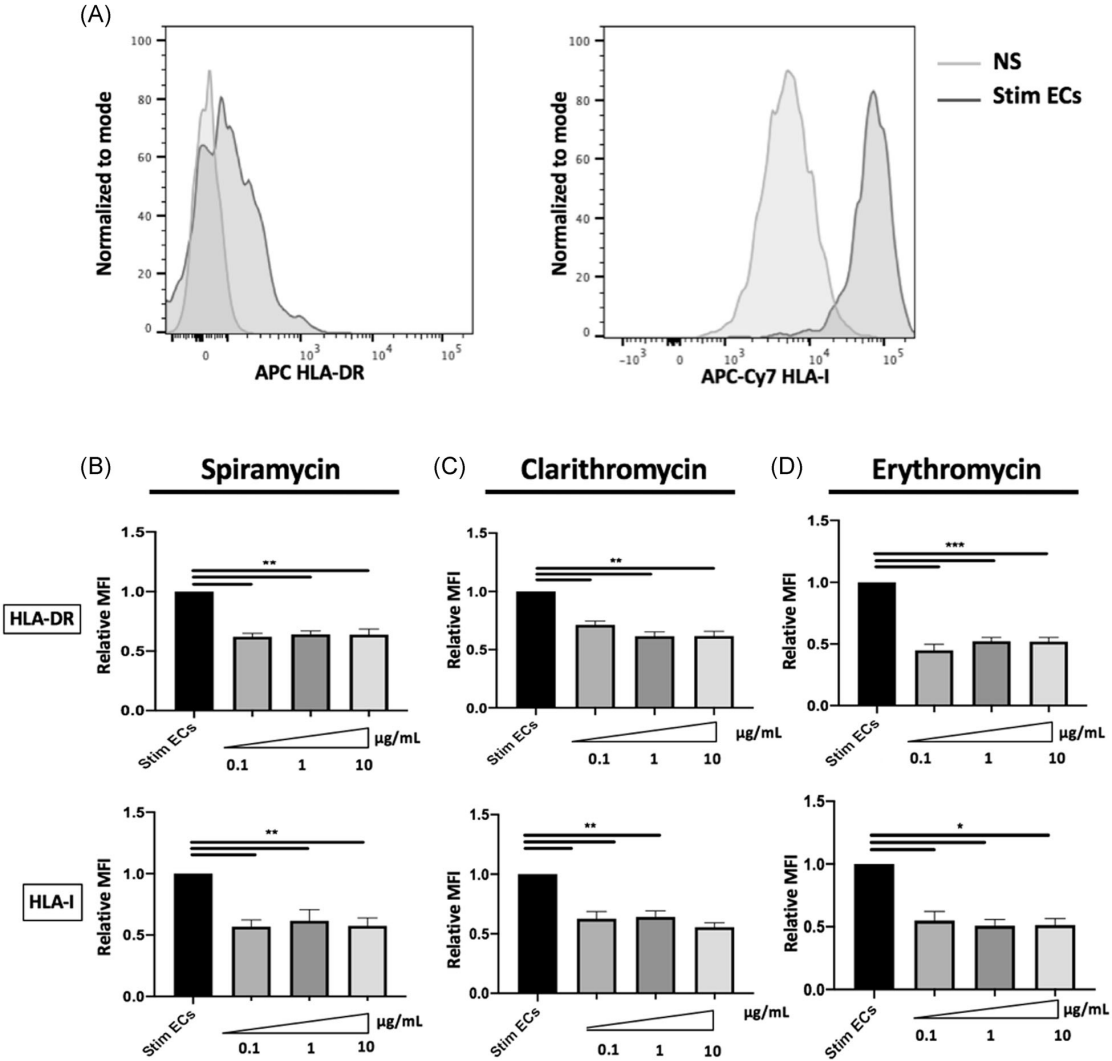
HLA‐expression by endothelial cells is decreased in the presence of macrolides. The human dermal microvascular endothelial cells (HMEC‐1) phenotype was assessed by flow cytometry after 24 h of septic stimulation by Interferon‐γ (IFN‐γ), tumor necrosis factor‐α (TNF‐α), and lipopolysaccharide (LPS). Septic stimulation induced a significant increase of HLA‐DR and HLA‐I expression on ECs. Panel A shows typical flow cytometry profiles (normalized to mode Mean Fluorescence Intensity [MFI]) of HLA‐DR and HLA‐I expressed by nonstimulated ECs (NS) and by septic stimulated ECS (Stim ECs). Spiramycin, Clarithromycin, or Erythromycin modified the cell‐surface phenotype of septic‐stimulated human ECs. The ECs phenotype was assessed after 24 h of septic stimulation by IFN‐γ, TNF‐α, and LPS, followed by 24 h’ incubation with different doses of Spiramycin (B), Clarithromycin (C), or Erythromycin (D). Control values for septic‐stimulated ECs incubated with the vehicle solution are represented as Stim ECs. The relative MFIs of HLA‐DR and HLA‐I are shown after treatment by Spiramycin (*n* = 3), Clarithromycin (*n* = 5), or Erythromycin (*n* = 3). The MFI is expressed relative to the MFI expressed by the stimulated ECs alone. The mean ± SEM (*p  <  .05, **p  <  .01, and ***p  <  .001, Kruskal–Wallis test) are shown. EC, endothelial cells; HLA, human leukocyte antigen; MFI, mean fluorescence intensity

We first examined the effect of macrolides on EC expression of HLA class I and class II molecules. Septic stimulation induced a significant increase of HLA‐DR and HLA‐I expression on HMEC‐1 (Figure 1A). Following incubation with different concentrations of Clarithromycin, Spiramycin, or Erythromycin (100ng–10 μg/ml), a significant decrease of HLA‐DR and HLA‐I expression was observed compared with septic HMEC‐1 alone (Figure 1B−D). Indeed, in septic HMEC‐1, 1 μg/ml of Spiramycin decreased the expression of HLA‐I (relative Mean Fluorescence Intensity (MFI) compared with stimulated ECs (Mean 0.62 [±SEM 0.09] vs. 1.0 [±0.0] *p* < .01) and HLA‐DR (relative MFI to stimulated ECs Mean 0.64 [±SEM 0,.03] vs. 1.0 [(±0.0] *p* < .01).

We also examined the effects of macrolides on the hCMEC/D3 ECs line, a recognized strong model of human blood–brain barrier ECs (BBB).^26^ Similarly to HMEC‐1, septic stimulation increased the expression of HLA‐DR and HLA‐I expression on the ECs surface (Figure S2A). However, Spiramycin and Erythromycin did not induce any significant decrease of HLA‐DR nor HLA class I surface expression in stimulated hCMEC/D3 (Figure S2B‐C).

**Figure 2:**
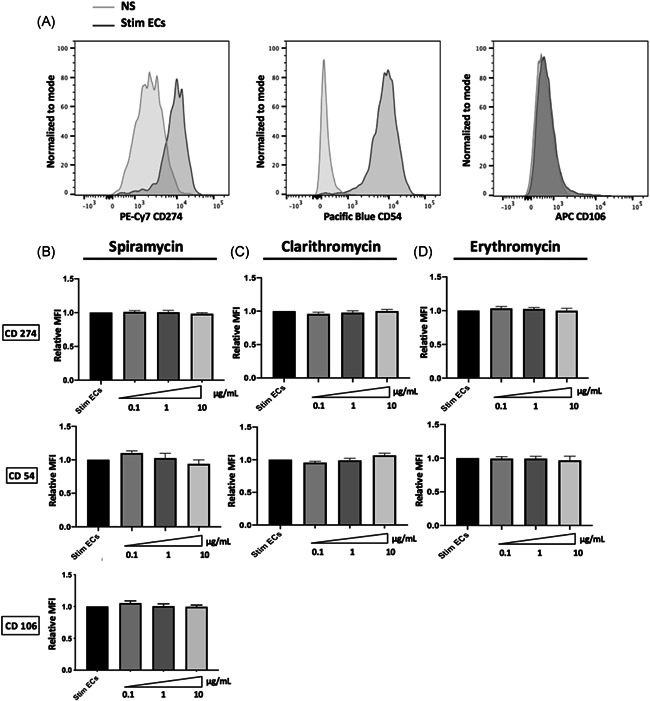
Interferon‐γ induced the expression of CD274, CD54, or CD106 is not modified by macrolides. The ECs phenotype was assessed by flow cytometry after 24 h of septic stimulation by IFN‐γ, TNF‐α and LPS. Septic stimulation induced a significant increase of CD54 and CD274 expression on ECs. Panel A shows a representative flow cytometry profile of CD54 and CD274 (normalized to mode Mean Fluorescence Intensity [MFI]) on nonstimulated ECs (NS) and on septic‐stimulated ECs (Stim ECs). Incubation of septic‐stimulated human microvascular ECs with macrolides did not alter Interferon‐γ induced expression of CD54 or CD274. The ECs phenotype was assessed after 24 h of septic stimulation by IFN‐γ, TNF‐α and LPS, followed by 24 h’ incubation with different doses of Clarithromycin (B), Spiramycin (C), or Erythromycin (D). Control values for ECs incubated with the vehicle solution are represented as Stim ECs. The relative MFIs of CD54 and CD 74 are shown after treatment by Clarithromycin (*n* = 5); Spiramycin (*n* = 3) or Erythromycin (*n* = 3). The MFI is expressed relative to the MFI expressed by the stimulated ECs alone. The mean ± SEM (*p  <  .05, **p  <  .01, and ***p  <  .001, Kruskal−Wallis test) are shown. EC, endothelial cell; IFN‐γ, Interferon γ; LPS, lipopolysaccharide; TNF‐α,tumor necrosis factor‐α

To further investigate the effects of macrolides on molecules upregulated during sepsis, we studied surface expression of CD54 (intercellular adhesion molecule [ICAM]‐1), CD106 (vascular cell adhesion molecule‐1) involved in leukocytes adhesion and CD274 (PDL‐1) a T‐lymphocyte inhibitor costimulatory molecule. In both ECs types, septic stimulation induced a significant increase of CD54, CD106, and CD274 molecules surface expression (Figures 2A and S2A). Macrolides did not induce any significant change in CD54, CD106, or CD274 expression on septic‐stimulated HMEC‐1 (Figure 2B−D) and on hCMEC/D3 (Figure S2D−F).

### 3.2 Macrolide therapy has no effect on septic‐stimulated ECs proinflammatory cytokines production

**Figure 3:**
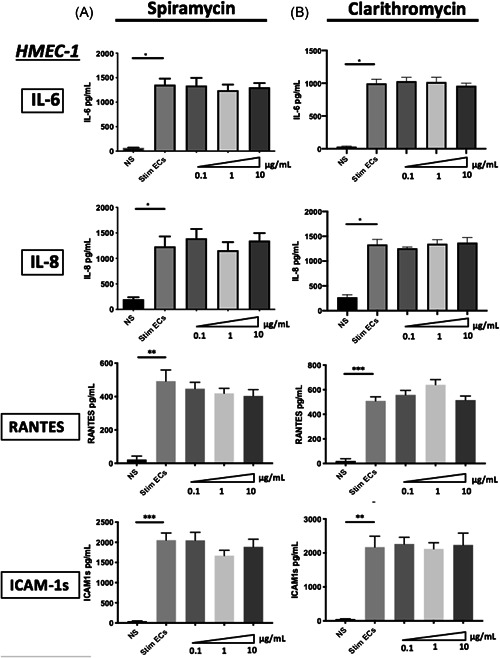
Effects of macrolide therapy on proinflammatory soluble factors production by septic stimulated human microvascular endothelial cells. Interleukin (IL)‐6, IL‐8, RANTES, and ICAM‐1s production were quantified by ELISA in the supernatants of human microvascular ECs (HMEC‐1). IL‐6, IL‐8, RANTES, and ICAM‐1s production was significantly increased after 24 h of stimulation by IFN‐γ, TNF‐α, and LPS compared with nonstimulated ECs (A,B). Treatment with Spiramycin (A) (*n* = 3) or Clarithromycin (B) (*n* = 3) for 24 h following septic stimulation did not significantly alter either IL‐6, IL‐8, RANTES, or ICAM‐1s production by septic‐stimulated ECs. Control values for nonstimulated ECs are represented as NS. Septic‐stimulated ECs are represented as Stim ECs. The mean ± *SEM* (*p  <  .05, **p  <  .01, and ***p  <  .001, Kruskal–Wallis test) are shown. EC, endothelial cell; ELISA, enzyme‐linked immunosorbent assay; IL‐6, interleukin‐6; ICAM‐1s, Inter Cellular Adhesion Molecule‐1

To investigate the impact of macrolides therapy on ECs activation, we examined the production of proinflammatory soluble factors by septic‐stimulated HMEC‐1 and hCMEC/D3 either treated or not by macrolides. We have examined IL‐6, IL‐8, ICAM‐1s, and RANTES production by ECs, all of which are implicated in immune regulation. Septic stimulation induced a significant increase of IL‐6 (from 65.4 pg/ml (±7.11) to 1355 pg/ml (±124.7) p < .001), IL‐8 (197.1 pg/ml (±40.93) to 1232 pg/ml (±197.2) *p* < .001), RANTES (19.63 pg/ml (±19.63) to 508.8 pg/ml (±33.37) *p* < .01)) and ICAM‐1s (52.96 pg/ml (±11.48) to 2171 pg/ml (±320.6) *p* < .01) by septic HMEC‐1 compared with nonstimulated ECs (Figure 3A,B). The same increase of proinflammatory factors production was also observed in hCMEC/D3 (Figure S3A,B). In HMEC‐1 and hCMEC/D3, treatment with different macrolides did not change production of the above factors. Indeed, IL‐6 secretion was stable in 1 μg/ml Spiramycin‐treated septic‐stimulated HMEC‐1 (1242 pg/ml [±117.4]) compared with septic‐stimulated HMEC‐1 (1355 pg/ml [±124.7] *p* = .87), as was IL‐8, RANTES and ICAM‐1s secretion (Figure 3A). The same results were found with stimulated hCMEC/D3 ECs treated by macrolides (Figure S3A,B).

### 3.3 Clarithromycin induces decreased IL‐6 gene expression but does not alter HLA‐A and HLA‐DR gene expression

**Figure 4:**
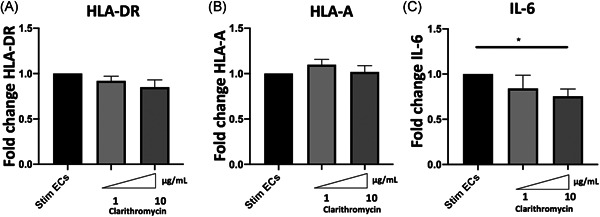
Impact of Clarithromycin on septic‐stimulated human microvascular endothelial cells (ECs) gene expression. The transcription of HLA‐DR, HLA‐A, and IL‐6 in human microvascular dermal ECs (HMEC‐1) was examined by qRT‐PCR after 24 h of septic stimulation by IFN‐γ, TNF‐α, and LPS, followed by 24 h’ incubation with different doses of Clarithromycin. The results are expressed as fold change of septic‐stimulated ECs gene expression. The relative transcription levels of HLA‐A, HLA‐DR, and IL‐6 are shown after treatment of septic‐stimulated ECs with Clarithromycin (*n* = 5). Clarithromycin did not induce any change in HLA‐DR (A) nor in HLA‐A (B) gene expression. However, Clarithromycin 10 μg/ml significantly decreased IL‐6 (C) gene expression in septic HMEC‐1. For all graphs, the mean  ±  SEM are indicated (*p  <  .05, **p  <  .01, and ***p  <  .001, unpaired *t* test). IL‐6, Interleukin‐6; IFN‐γ, Interferon γ; LPS, lipopolysaccharide; qRT‐PCR, quantitative real‐time polymerase chain reaction; TNF‐α,tumor necrosis factor‐α

To further explore the mechanisms responsible for HLA class I and HLA‐DR decreased the expression in stimulated ECs, we next determined whether the changes in protein expression were related to modifications of mRNA expression. Clarithromycin did not induce any change in HLA‐A and HLA‐DR genes expression (Figure 4A,B). However, 10 μg/ml Clarithromycin significantly decreased IL‐6 gene expression in septic‐stimulated HMECs (relative transcription level of IL‐6 to stimulated ECs 0.75 [±0.18] vs. 1.0 [±0] *p* < .02) (Figure 4C).

### 3.4 Phenotypic changes in ECs do not induce T‐lymphocyte mortality

**Figure 5:**
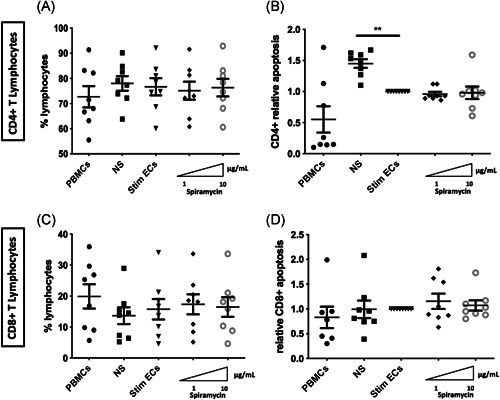
Effects of Spiramycin on the viability of septic‐stimulated ECs cocultured with peripheral blood mononuclear cells. Proportion of CD4 ^+ ^and CD8 ^+ ^lymphocytes and T‐lymphocyte viability were studied after a 3‐day incubation with septic‐ stimulated human dermal microvascular ECs (HMEC‐1) in the presence or absence of Spiramycin. The proportion of CD4 ^+ ^and CD8 ^+ ^lymphocytes was unchanged by Spiramycin (*n* = 8 donors) (A−C). Apoptosis of CD4 ^+ ^T or of CD8 ^+ ^T lymphocytes, was unaltered by exposure to Spiramycin (*n* = 8 donors) (B−D). Control values for nonstimulated ECs in coculture with PBMCs are represented as NS and PBMCs alone are represented as PBMCs. Septic‐stimulated ECs in coculture with PBMCs are represented as Stim ECs. Relative apoptosis is expressed as apoptosis of T lymphocytes in PBMC cocultured with septic‐stimulated ECs. The mean ± SEM (*p  <  .05, **p  <  .01, and ***p  <  .001, Repeated measure one‐way ANOVA test) are shown. ANOVA, analysis of variance; EC, endothelial cell; PBMCs, peripheral blood mononuclear cells

In sepsis, it has been demonstrated that lymphocyte experiment accelerated apoptosis, playing a central role in secondary infections and in late death of septic patient.^28^, ^29^ Therefore, we evaluated whether decreased expression of HLA induced by macrolides in stimulated ECs was responsible for T lymphocyte mortality by determining the proportion of CD4 ^+ ^and CD8 ^+ ^‐T lymphocytes as well as cell death after 3 days of coculture of PBMCs and septic‐stimulated HMEC‐1. Compared with PBMCs alone, the proportions of CD4 ^+ ^and CD8 ^+ ^‐T lymphocytes were unchanged in cocultures (Figure 5A−C). Moreover, compared with nonstimulated ECs in coculture with PBMCS, septic‐stimulated ECs significantly decreased CD4 ^+ ^‐T lymphocyte apoptosis (relative apoptosis 1.45 [±0.07] vs. 1.0 [±0] *p* < .01) (Figure 5B). However, incubation of septic‐stimulated ECs with Spiramycin before coculture with PBMCs did not alter either CD8 ^+ ^or CD4 ^+ ^‐T lymphocyte proportions or mortality compared with control stimulated ECs (Figure 5A−D).

### 3.5 Phenotypic changes in ECs do not result in modification of T‐ lymphocyte polarization

**Figure 6:**
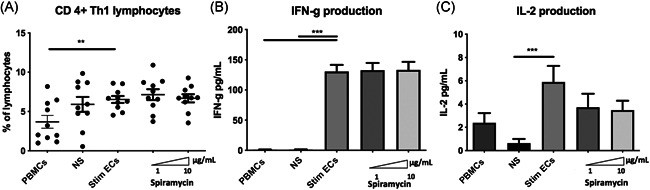
Spiramycin did not alter differentiation of Th1 nor IL‐2 and IFN‐γ production in septic‐stimulated ECs‐PBMCs cocultures. Septic‐stimulated human dermal microvascular ECs (HMEC‐1) cocultured for 7 days with PBMCs activated differentiation of CD3 ^+ ^CD8^neg^IFN‐**γ** ^**+**^‐T lymphocytes (Th1) (A). Differentiation of Th1 lymphocytes was unaltered by Spiramycin (*n* = 10 donors) (A). IFN‐γ and IL‐2 production by Th1 was quantified, both were detected in coculture supernatants and increased by coculture of PBMCs with septic‐stimulated ECs (B,C). Spiramycin (*n* = 10 donors) did not alter IFN**‐**γ production (B). IL‐2 (C) levels were not significantly lessened by macrolide therapy. Control values for nonstimulated ECs in coculture with PBMCs are represented as NS and PBMCs alone are represented as PBMCs. Septic‐stimulated ECs cocultured with PBMCs are represented as Stim ECs. The mean ± SEM (*p  <  .05, **p  <  .01, and ***p  <  .001, Repeated measure one‐way ANOVA or Friedman test) are shown. ANOVA, analysis of variance; IL‐2, interleukin‐2; IFN‐γ, interferon‐γ; PBMCs, peripheral blood mononuclear cells

The Th1 lymphocyte subset is an important mediator of cell‐mediated immunity and phagocyte‐dependent protective responses. In this model of allogeneic CD4 ^+ ^‐T lymphocytes activation by ECs, Th1 differentiation was increased by septic‐stimulated ECs compared with PBMCs alone (gating strategy shown in Figure S4) (Figure 6A). However, stimulated ECs treated by different doses of Spiramycin did not significantly change proinflammatory CD3 ^+ ^CD8^neg^IFN‐γ ^+^ Th1 cells differentiation compared with stimulated ECs alone (Figure 6A). In addition to intracellular cytokine staining, we also determined IL‐2 and IFN‐γ production by ELISA assays in coculture supernatants. Spiramycin did not alter IFN**‐**γ production and IL‐2 levels were not significantly lessened by macrolide therapy (Figure 6B,C).

**Figure 7:**
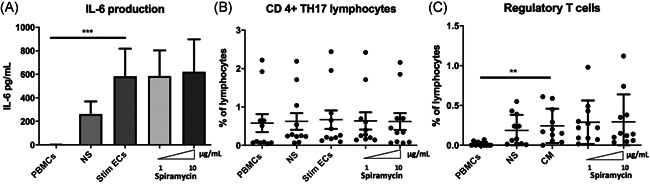
Spiramycin did not alter IL‐6 production nor Treg or Th17 lymphocytes differentiation in septic‐stimulated ECs‐PBMCs cocultures. IL‐6 production was assessed by ELISA in the supernatants of PBMCs‐septic‐stimulated human microvascular dermal ECs (HMEC‐1) cocultures after 3 days. IL‐6 production was significantly increased by stimulated ECs in coculture with PBMCs compared with PBMCs alone. However, macrolide therapy did not alter IL‐6 production in PBMCs‐stimulated ECs cocultures (*n* = 11 donors) (A). Moreover, Spiramycin pretreatment of septic‐stimulated microvascular ECs cocultured for 7 days with PBMCs did not alter CD3 + CD8negIL17 + Th17 lymphocytes polarization (B). Septic‐stimulated ECs cocultured for 7 days with PBMCs activated differentiation of Treg compared with PBMCs alone (C). Differentiation of the Treg population was unaltered by Spiramycin (*n* = 11 donors) (C). Septic‐stimulated ECs in coculture with PBMCs are represented as Stim ECs. The mean ± SEM (*p  <  .05, **p  <  .01, and ***p  <  .001, Friedman test) are shown. ELISA, enzyme‐linked immunosorbent assay; ECs, endothelial cells; IL‐6, interleukin‐6; PBMCs, peripheral blood mononuclear cells

ECs expression of HLA‐DR and CD54 molecules has been reported to be required for amplification of regulatory T lymphocytes (Tregs), whereas expansion of the Th17 subset is dependent on endothelial secretion of IL‐6.^18^ IL‐6 is also known as an inhibitor of Foxp3 expression and function and therefore contributes to controlling the balance between Th17 and Treg.^30^ We measured IL‐6 in coculture supernatants to determine whether it was changed by the decreased HLA‐DR expression on stimulated ECs in the presence of macrolides. IL‐6 production was significantly increased in cocultures of PBMCs and septic‐ stimulated ECs (583 pg/ml [±236]) compared with PBMCs alone (0.87 pg/ml (±0.87) *p* < .01) (Figure 7A). Th17 lymphocytes have been strongly involved in protection against bacterial and fungal pathogens through production and induction of inflammatory cytokines and are also involved in the granulopoiesis, and the recruitment of neutrophils.^31^, ^32^ Exposure of septic‐ stimulated HMEC‐1 to Spiramycin did not alter IL‐6 production or Th17 expansion (gating strategy in Figure S4) (Figure 7B). The Treg population is defined as CD4 ^+ ^CD45RA^neg^FoxP3^bright^ (gating strategy shown in Figure S4), and are also CD25 ^+ ^and CD127^low^. Differentiation of Treg was significantly activated by septic ECs (percentage of lymphocytes in PBMCs 0.24% [±0.07%]) compared with PBMCs alone (0.02% (±0.01%) *p* < .05). However, the proportion of Treg was not altered by macrolides under the same conditions (Figure 7C).

## 4 DISCUSSION

This is the first study of the immunomodulatory effects of macrolides on septic‐stimulated microvascular human ECs. Although a significant decrease in cell‐surface HLA‐I and HLA‐DR expression and in IL‐6 gene expression was observed, these modifications did not result in altered functional response following EC interaction with PBMCs regarding T‐ lymphocyte viability, cytokine production, or CD4^+ ^‐T differentiation.

In addition to their antimicrobial activity, numerous previous studies have suggested that macrolides may exert immunomodulatory properties.^33^ Some of these properties were highlighted in airway epithelial cells. Thus, macrolide therapy can decrease mucus secretion,^34^, ^35^ adhesion of neutrophils to bronchial epithelial cells^36^, ^37^ and can increase the barrier function of airway epithelium.^38^, ^39^ In vivo, in a mouse model of CAP, it has been shown that macrolides associated with B‐lactam increased survival but could also act directly on immune cells leading to significant phenotypic modifications of neutrophils resulting in altered Major Complex Histocompatibility class II and decreased cytotoxic‐T lymphocyte‐associated antigen 4 and programmed death 1 on CD4^+^ ‐T and Treg.^10^

Because of their potential immunoregulating properties associated with their activity against atypical intracellular pathogens, macrolides are frequently used in critically ill patients with severe CAP. However, in a recent clinical study of 7182 critically ill patients with acute respiratory failure we did not find any association between macrolide therapy and survival, mechanical ventilation duration or secondary infection acquisition in critically ill patients with acute respiratory failure.^40^ Nevertheless, other studies of smaller patient cohorts had suggested that macrolide therapy may have some beneficial effects due to their immunomodulatory properties in intensive care patients.^12^, ^41^ This discrepancy may be explained by differences in the sizes of the cohorts, the statistical models used, statistical power stemming from a larger patient group, as well as variability in the timing and doses of macrolides administered.

We chose to use the HMEC‐1 cell line in this study to explore the potential immune effects of macrolides on ECs. This microvascular EC line provides a robust model of human microvascular ECs due to (i) its steady‐state secretion of endothelial‐associated soluble factors already identified in vivo (e.g., IL‐6 and MCP‐1), (ii) a steady‐state expression of low levels of HLA‐I and CD274 that are highly increased in the presence of proinflammatory factors, and (iii) the expression of the costimulatory molecules OX‐40L, 4IBB‐L, and low levels of ICOS‐L. Microvascular ECs express a low level of CD54 and HLA‐DR in vivo and HLA‐DR expression is progressively lost in in vitro culture in the absence of proinflammatory factors.^42^ HMEC‐1 is therefore an imperfect model regarding steady‐state CD54 and HLA‐DR although the high increase in their expression in the presence of IFN‐γ, as well as the further increase when both IFN‐γ and TNF‐α are present, reproduce the higher expression level of both molecules under inflammatory conditions in vivo.^19^, ^43^ In addition to the phenotype and soluble factor secretion, HMEC‐1 also reproduces functional roles of human ECs regarding its ability for memory CD4 ^+ ^‐T lymphocyte activation and differentiation into pro‐and anti‐inflammatory subsets under inflammatory conditions.^19^, ^44^ Finally, the use of a cell line permitted a high level of reproducibility between experiments.

In the current study, we did not find any effect of macrolide therapy on cytokines produced by ECs, namely, IL‐6 and IL‐8 that are secreted in high amounts under septic conditions in our model. IL‐8 is one of the cysteine‐X‐cysteine chemokines and is a potent neutrophil attractant. In previous studies, Azithromycin reduced IL‐8 concentration in BAL from patients with lung transplantation^45^ and was able, in vitro, to inhibit release of IL‐8 from macrophages and leucocytes.^46^ IL‐6 is a pleiotropic proinflammatory cytokine^47^ secreted by several different cell types of cardiovascular relevance, including macrophages, lymphocytes, fibroblasts, ECs and smooth muscle cells.^48^, ^49^, ^50^ In BAL from patient with cryptogenic organizing pneumonia, the concentration of IL‐6 decreased with Clarithromycin treatment.^51^ In vitro, Erythromycin (10 μg/ml) inhibited IL‐6 secretion of human bronchial epithelial cells stimulated by endotoxin.^52^ Macrolide treatment did not alter IL‐6 nor IL‐8 production by septic‐stimulated ECs. However, we cannot exclude that either the strong level of inflammation induced under these conditions may have masked a subtle effect of macrolide therapy on cytokine secretion by ECs or that longer exposure of ECs to macrolides may result in reduced IL‐6 as reported in clinical studies. Furthermore, because decreased IL‐6 gene expression was detected, this may indicate that reduced protein would be observed after longer periods of exposure to macrolides.

Beyond their role as passive target during sepsis, ECs are conditional antigen presenting cells. Microvascular ECs express HLA class II antigens in the steady‐state and expression is highly increased under inflammatory conditions.^17^, ^42^, ^53^ They allow, in some specific situations (inflammation, uncontrolled infections by the innate immune system), the initiation of adaptive immunity.^44^, ^54^ The decrease in cell‐surface HLA‐I and HLA‐DR on ECs induced by macrolides after septic stimulation led us to examine their role in adaptive immunity using a model of allogeneic antigen presentation. Interestingly, we found that Treg expansion was observed when ECs had been exposed to septic conditions. This amplification of Treg is likely to be due to the expression of HLA‐DR and CD54 induced by modeling septic conditions. Moreover, given that a key cytokine for Treg maintenance and survival is IL‐2, this may also be due to increased IL‐2 in cocultures of septic‐stimulated ECs compared with nonstimulated ECs. Previous clinical studies found that after the onset of septic shock, the number of Tregs was increased and that the relative increase in circulating Treg may be implicated in lymphocyte anergy described after septic shock.^55^, ^56^ In neonates, expansion of activated Treg inversely correlates with the severity of sepsis.^57^ Little is known about the effects of macrolide therapy on Treg differentiation in a septic context. Only one study analyzed the effects of macrolide therapy on Treg in a mouse model of chronic *Pseudomonas aeruginosa* lung infection. In this article, low‐dose Clarithromycin induced a significant downregulation of the Treg response.^58^ In ECs, HLA‐DR is necessary and sufficient for T‐ cell proliferation and Treg expansion in our coculture model.^19^ However, we did not observe any effect of macrolides on antigen presentation by human ECs in a septic environment. One possibility is that the production of IFN‐γ in the coculture setting (but not by ECs alone), in the presence or absence of macrolides, overrides the ability of macrolides to decrease HLA‐DR and that the decrease observed in cultures of ECs is not reproduced in the coculture setting. Indeed, the increase in IFN‐γ (and of IL‐2) is particularly marked in cocultures of the septic‐stimulated EC compared with nonactivated ECs.

The Th1 subset was amplified by sepsis‐stimulated ECs but was not altered by the presence of macrolides. This result is in accordance with IL‐6 production in our model of coculture as Th1 dependence on IL‐6 produced by ECs has been reported in vitro and in vivo.^19^, ^44^

Moreover, although the model benefits from studying primary circulating leukocyte responses, it is hampered by examining antigen presentation in an allogeneic context. However, allogeneic stimulation is frequently tested as a model for T‐lymphocyte activation by HLA class II molecules when the peptide has not been identified and the antigen is an unknown peptide associated with a non‐self HLA molecule in this setting. The number of PBMC donors used in each experimental setting allowed appropriate analysis of the responses obtained. The possibility of obtaining HLA‐matched ECs and PBMCs is not currently available and the possible use of murine ECs would also be limited given the documented differences in their immunological characteristics compared with human cells.^59^

Finally, primary ECs of different origins are remarkably heterogeneous in terms of gene expression, antigen composition, and function.^60^ Studying two different microvascular cells, we indeed showed that HLA class I and HLA‐DR expressions were different under septic condition. We cannot exclude that results would have been different using other ECs, whether macrovascular or microvascular ECs.

## 5 CONCLUSION

This study reports phenotypic and gene expression changes in septic‐stimulated microvascular ECs after exposure to macrolides. Although a significant decrease in cell‐surface HLA class I and HLA‐DR expression and in IL‐6 gene expression was observed, these modifications did not result in altered functional response following ECs interaction with PBMCs regarding T lymphocyte viability, cytokine production or CD4 ^+ ^‐T differentiation. *In vivo* studies may help us to further investigate the immune consequences on ECs during macrolides therapy.

## CONFLICT OF INTERESTS

The authors declare that there is no conflict of interest.

## AUTHOR CONTRIBUTIONS

*Experimental concept and design*: Stéphanie Pons, Eden Arrii, Lara Zafrani, and Nuala Mooney. *Performance/realization of the experiments*: Stéphanie Pons, Eden Arrii, Marine Arnaud, Maud Loiselle, Juliette Ferry, Manel Nouacer, Julien Lion, and Shannon Cohen. *Contribution of reagents, materials, and analysis tools*: Stéphanie Pons, Lara Zafrani, and Nuala Mooney. *Data analysis*: Stéphanie Pons, Eden Arrii, Lara Zafrani, and Nuala Mooney. *Writing of the paper*: Stéphanie Pons, Eden Arrii, Lara Zafrani, and Nuala Mooney. All authors contributed to the article and approved the submitted version.

## Supporting information

Supplementary information.

Supplementary information.

Supplementary information.

Supplementary information.

Supplementary information.

## Data Availability

The data that support the findings of this study are available from the corresponding author upon reasonable request.
