# Supplementary material for: Immunomodulation of endothelial cells induced by macrolide therapy in a model of septic stimulation
Source: Immun Inflamm Dis. 2021 Oct 12;9(4):1656–69. doi: 10.1002/iid3.518 (PMC8589380; doi:10.1002/iid3.518)
Supplement: Supplementary file 1 — Supplementary information. [file IID3-9-1656-s004.docx]

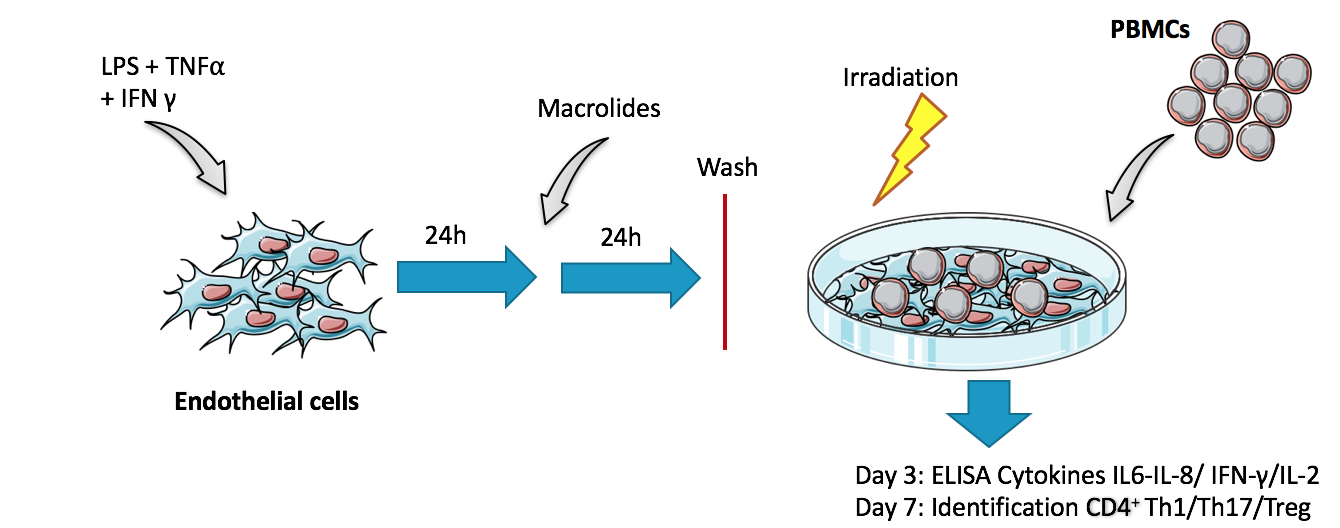


**Supplemental Figure 1: Co-culture of endothelial cells and peripheral blood mononuclear cells**

After 24 hours of stimulation by lipopolysaccharide, tumor necrosis factor α and interferon γ, endothelial cells were either exposed to macrolides or to control conditions for another 24 hours. Endothelial cells were then washed three times to remove septic stimulation. Cells were irradiated (20 Gy) to prevent further proliferation and co-cultured with PBMCs at a ratio 1:1 for 7 days. The supernatants of co-cultures were collected after 72 hours for cytokines measurement (interleukin (IL)-6, IL-8, IFN-γ and IL-2). At day 7 of the co-culture, PBMCs were stimulated by phorbol-12-myristate-13-acetate 50 ng/ml, and ionomycin 1 µM in the presence of GolgiStop for four hours before labeling cells to detect T lymphocytes expressing intracellular IL-17 (CD3^+^CD8^−^IL-17^+^) or IFN-γ (CD3^+^CD8^−^IFN-γ^+^) by flow cytometry.
