# Supplementary material for: Immunomodulation of endothelial cells induced by macrolide therapy in a model of septic stimulation
Source: Immun Inflamm Dis. 2021 Oct 12;9(4):1656–69. doi: 10.1002/iid3.518 (PMC8589380; doi:10.1002/iid3.518)
Supplement: Supplementary file 2 — Supplementary information. [file IID3-9-1656-s001.docx]

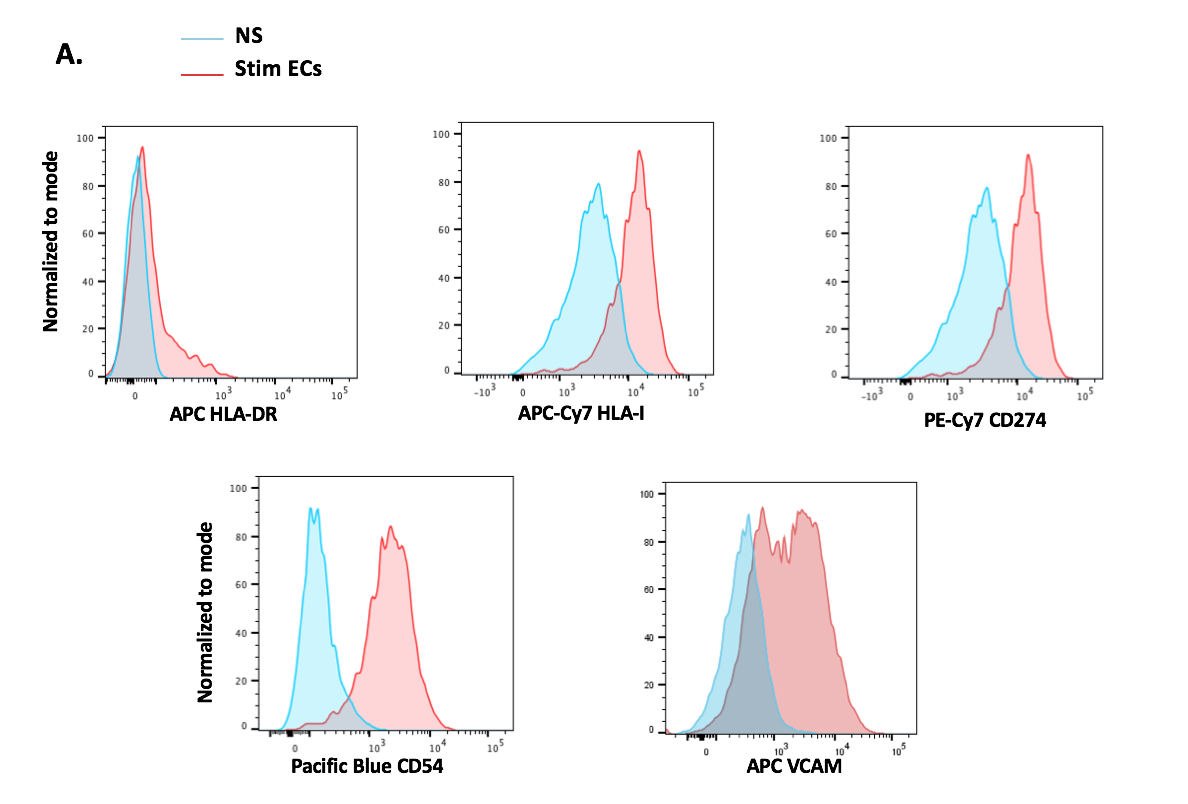


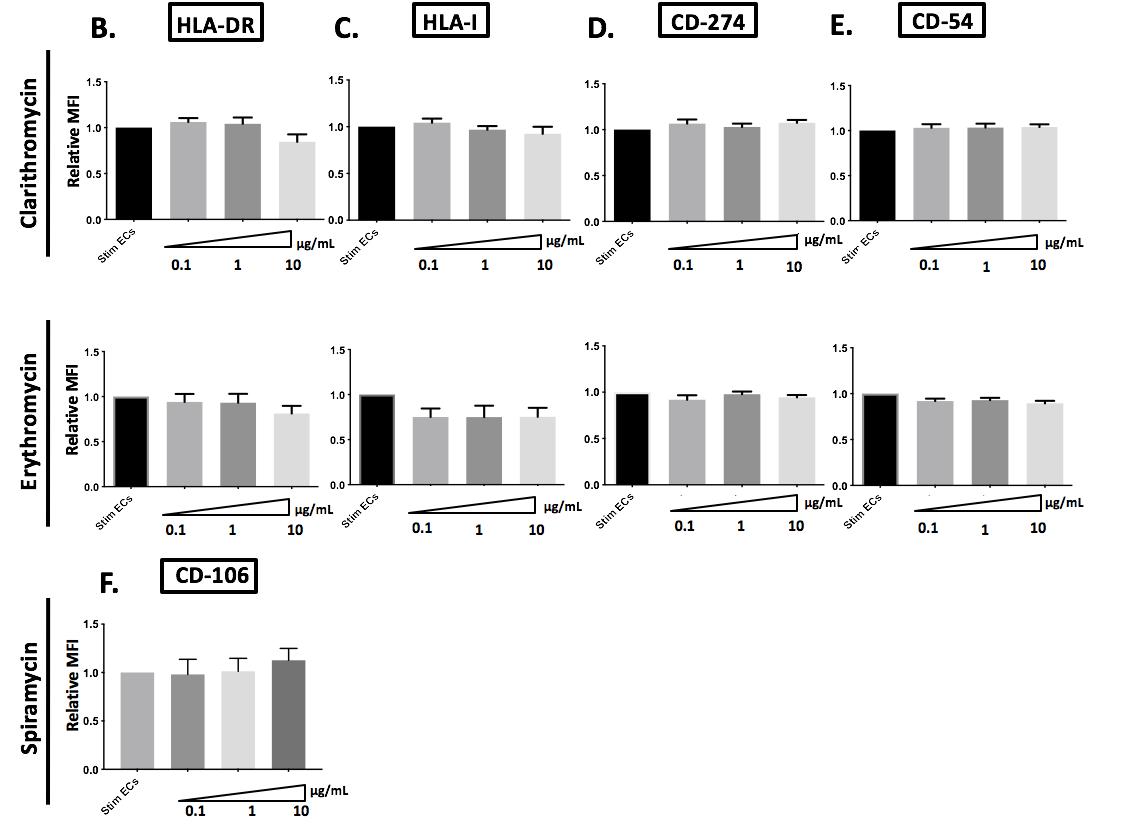


**Supplemental Figure 2**

**Clarithromycin, Erythromycin or Spiramycin did not modify the phenotype of septic stimulated human blood brain barrier endothelial cells (ECs).**

The blood brain barrier ECs phenotype was assessed after 24 hours of septic stimulation by IFN-γ, TNF-α and LPS. Septic stimulation induced a significant increase of HLA-DR, HLA-I, CD54, CD106 and CD274 expression on ECs. Panel A shows typical flow cytometry profiles (normalized to mode Mean Florescence Intensity (MFI)) of HLA-DR, HLA-1, CD54, CD106 and CD274 on non-stimulated ECs (NS) and on septic stimulated ECS (Stim ECs).

Incubation of septic stimulated human microvascular blood brain ECs with macrolides did not alter the expression of HLA-DR, HLA-I, CD54, CD106 or CD274 on septic ECs. The ECs phenotype was assessed after 24 hours of septic stimulation by IFN-γ, TNF-α and LPS, followed by 24 hours of incubation with Clarithromycin, Erythromycin or Spiramycin. Control values for ECs incubated with vehicle solution are shown (Stim ECs). The relative MFIs of HLA-DR (B), HLA class I (C), CD274 (D), CD54 (E) and CD106 (F) are shown after treatment by Clarithromycin (n=4), Erythromycin (n=5) or Spiramycin (n=4). The MFI is calculated relative to the MFI expressed by the stimulated ECs alone. The mean ± SEM (**p* < 0.05, ***p* < 0.01, and ****p* < 0.001, Kruskal-Wallis test) are shown.
