# Supplementary material for: Immunomodulation of endothelial cells induced by macrolide therapy in a model of septic stimulation
Source: Immun Inflamm Dis. 2021 Oct 12;9(4):1656–69. doi: 10.1002/iid3.518 (PMC8589380; doi:10.1002/iid3.518)
Supplement: Supplementary file 3 — Supplementary information. [file IID3-9-1656-s002.docx]

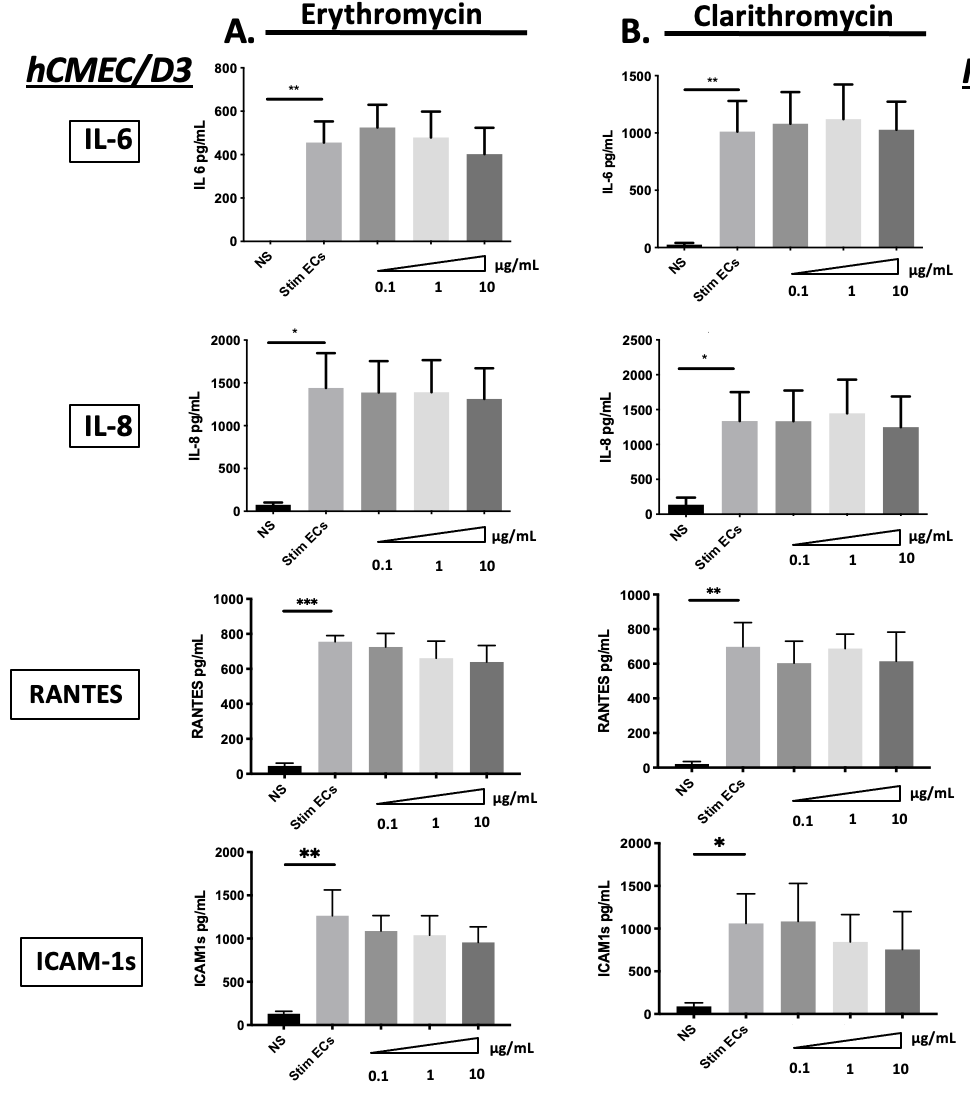


**Supplemental Figure 3**

**Effects of macrolide therapy on pro-inflammatory soluble factors production by human blood brain barrier endothelial cells (ECs)**

Interleukin (IL)-6, IL-8, RANTES and ICAM-1s production were quantified by ELISA in the supernatant of human blood brain barrier ECs (hCMEC/D3). IL-6, IL-8, RANTES and ICAM-1s were significantly increased after 24 hours of septic stimulation by IFN-γ, TNF-α and LPS (A-B). Treatment with Erythromycin (A) (n=3) and Clarithromycin (B) (n=4) in hCMEC/D3 for 24 hours following septic stimulation did not alter cytokines production by septic stimulated ECs. Control values for non-stimulated ECs are represented as NS and for septic stimulated ECs as Stim ECs. The mean ± SEM (*p < 0.05, **p < 0.01, and ***p < 0.001, One-way ANOVA) are shown.
