# Supplementary material for: Immunomodulation of endothelial cells induced by macrolide therapy in a model of septic stimulation
Source: Immun Inflamm Dis. 2021 Oct 12;9(4):1656–69. doi: 10.1002/iid3.518 (PMC8589380; doi:10.1002/iid3.518)
Supplement: Supplementary file 4 — Supplementary information. [file IID3-9-1656-s003.docx]

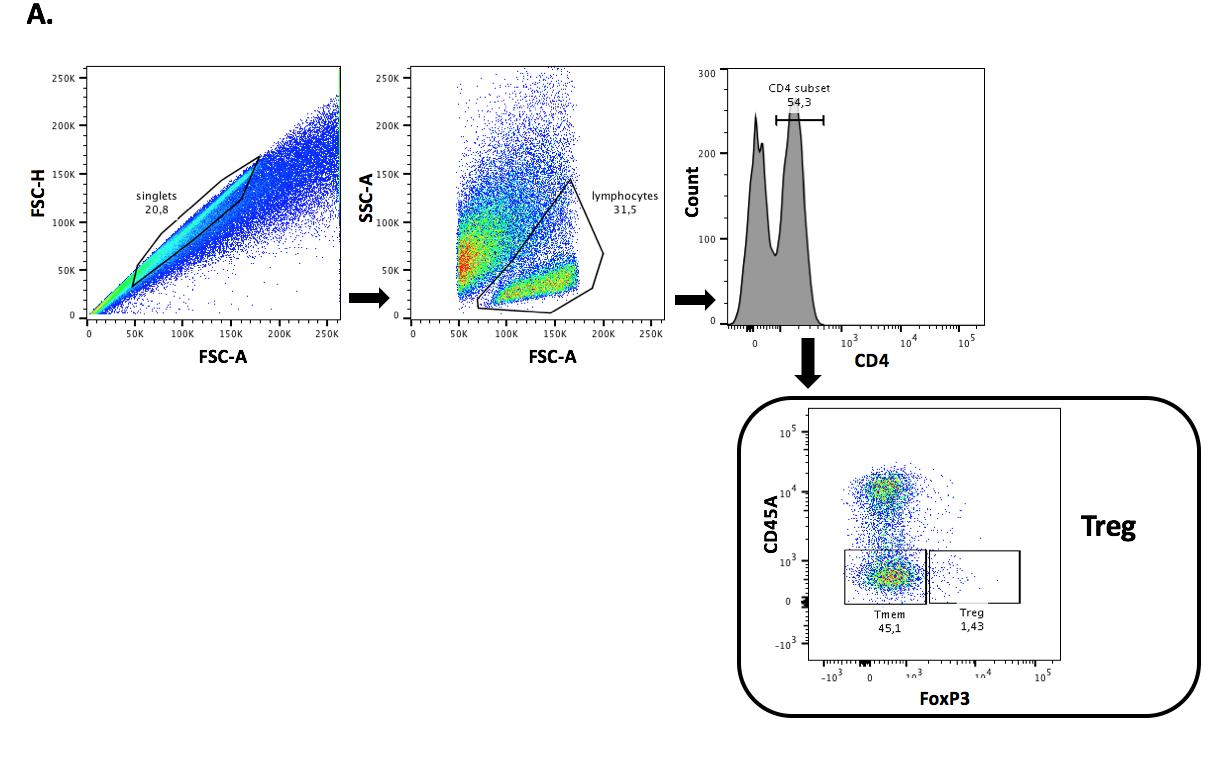


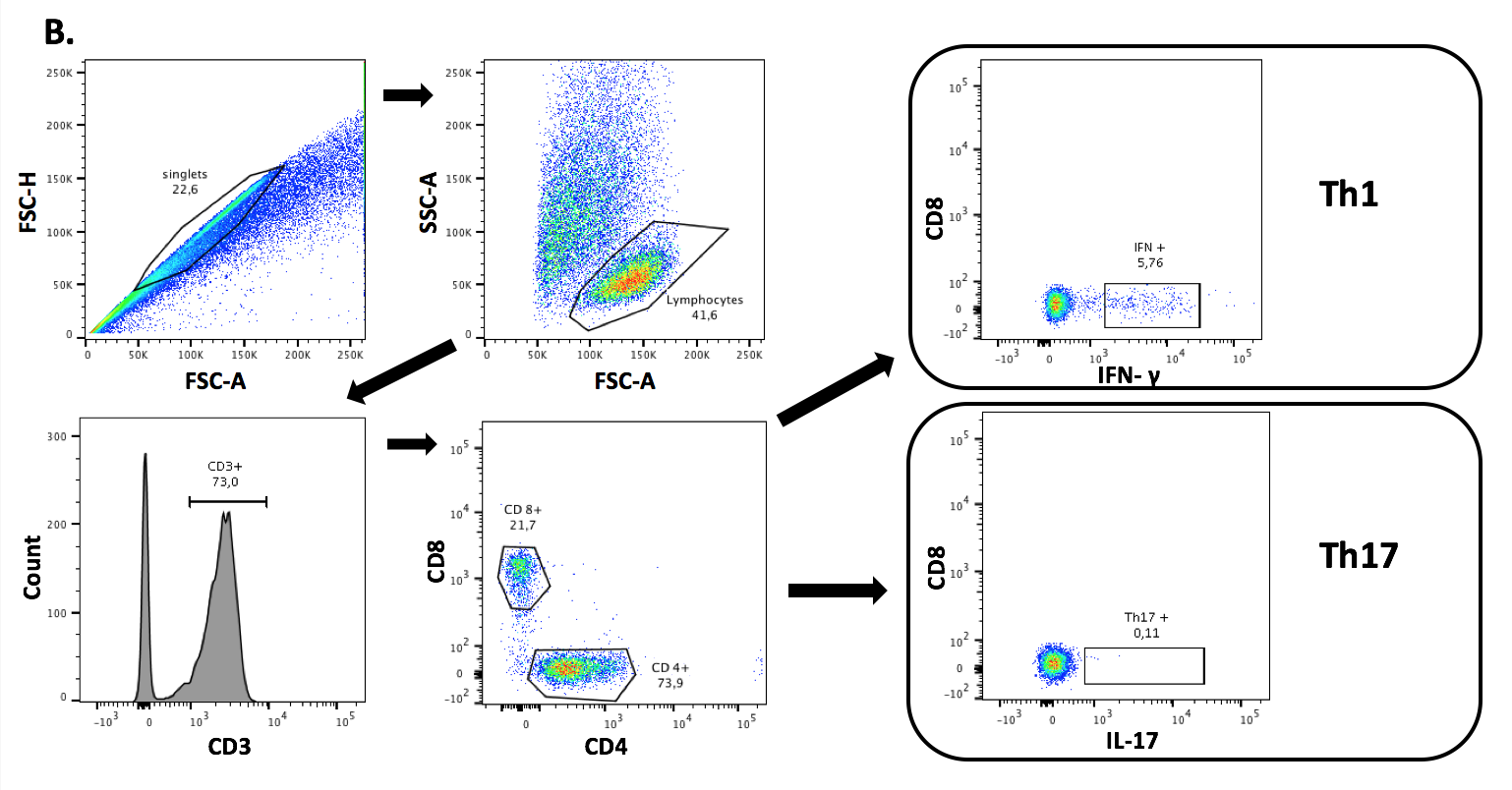


**Supplemental Figure 4**

**Gating strategy of Treg, Th1 and Th17 populations**

Figure S3A shows the gating strategy for identification of CD4^+^CD45RA^neg^FoxP3^high^ Treg cells after selection of single events. Figure S3B shows the gating strategy for identification of

CD3^+^CD8^neg^IFNγ^+^ Th1 cells and of CD3^+^CD4^+^CD8^neg^IL17^+^ Th17 cells after selection of single events.
