# Supplementary material for: Immunomodulation of endothelial cells induced by macrolide therapy in a model of septic stimulation
Source: Immun Inflamm Dis. 2021 Oct 12;9(4):1656–69. doi: 10.1002/iid3.518 (PMC8589380; doi:10.1002/iid3.518)
Supplement: Supplementary file 5 — Supplementary information. [file IID3-9-1656-s005.docx]

**Appendices**

**Supplemental** Figure 1: **Co-culture of endothelial cells and peripheral blood mononuclear cells**

After 24▒hours of stimulation by Lipopolysaccharide, Tumor Necrosis Factor α and Interferon γ, endothelial cells were either exposed to macrolides or not for a further 24▒hours. Endothelial cells were then washed three times to remove septic stimulation. Cells were irradiated (20▒Gy) to prevent further proliferation and co-cultured with PBMCs at a ratio 1:1 for 7 days. The supernatants of co-cultures were collected after 72▒hours for cytokines measurement (interleukin (IL)-6, IL-8, IFN-γ and IL-2). At day 7 of the co-culture, PBMCs were stimulated by phorbol-12-myristate-13-acetate 50▒ng/ml, and ionomycin 1▒µM in the presence of GolgiStop for four hours before labeling cells to detect T lymphocytes expressing intracellular IL-17 (CD3▒^+▒^CD8▒^−▒^IL-17^+^) or IFN-γ (CD3▒^+▒^CD8▒^−▒^IFN-γ ^+^) by flow cytometry.

**Supplemental** Figure 2: C**larithromycin, Erythromycin or Spiramycin did not modify the phenotype of septic stimulated human blood brain barrier endothelial cells (ECs).**

The blood brain barrier ECs phenotype was assessed after 24▒hours of septic stimulation by IFN-γ, TNF-α and LPS. Septic stimulation induced a significant increase of HLA-DR, HLA-I, CD54, CD106 and CD274 expression on ECs. Panel A shows typical flow cytometry profiles (normalized to mode Mean Florescence Intensity (MFI)) of HLA-DR, HLA-1, CD54, CD106 and CD274 on non-stimulated ECs (NS) and on septic stimulated ECS (Stim ECs).

Incubation of septic stimulated human microvascular blood brain ECs with macrolides did not alter the expression of HLA-DR, HLA-I, CD54, CD106 or CD274 on septic ECs. The ECs phenotype was assessed after 24▒hours of septic stimulation by IFN-γ, TNF-α and LPS, followed by 24▒hours of incubation with Clarithromycin, Erythromycin or Spiramycin. Control values for ECs incubated with vehicle solution are shown (Stim ECs). The relative MFIs of HLA-DR (B), HLA class I (C), CD274 (D), CD54 (E) and CD106 (F) are shown after treatment by Clarithromycin (n▒=▒4), Erythromycin (n▒=▒5) or Spiramycin (n▒=▒4). The MFI is calculated relative to the MFI expressed by the stimulated ECs alone. The mean▒±▒SEM (**p* ▒<▒ 0.05, ***p* ▒<▒ 0.01, and ****p* ▒<▒ 0.001, Kruskal-Wallis test) are shown.

**Supplemental** Figure [3](#MEP_L_fig3)**: Effects of macrolide therapy on pro-inflammatory soluble factors production by human blood brain barrier endothelial cells (ECs)**

Interleukin (IL)-6, IL-8, RANTES and ICAM-1s production were quantified by ELISA in the supernatant of human blood brain barrier ECs (hCMEC/D3). IL-6, IL-8, RANTES and ICAM-1s were significantly increased after 24▒hours of septic stimulation by IFN-γ, TNF-α and LPS (A-B). Treatment with Erythromycin (A) (n▒=▒3) and Clarithromycin (B) (n▒=▒4) in hCMEC/D3 for 24▒hours following septic stimulation did not alter cytokines production by septic stimulated ECs. Control values for non-stimulated ECs are represented as NS and for septic stimulated ECs as Stim ECs. The mean▒±▒SEM (*p ▒<▒ 0.05, **p ▒<▒ 0.01, and ***p ▒<▒ 0.001, One-way ANOVA) are shown.

**Supplemental** Figure 4: **Gating strategy of Treg, Th1 and Th17 populations**

Figure S3A shows the gating strategy for identification of CD4▒^+▒^CD45RA^neg^FoxP3^high^ Treg cells after selection of single events. Figure S3B shows the gating strategy for identification of CD3▒^+▒^CD8^neg^IFNγ▒^+▒^Th1 cells and of CD3▒^+▒^CD4▒^+▒^CD8^neg^IL17▒^+▒^Th17 cells after selection of single events.
